# Eosinophilic infiltration as the initial trace of acute mixed cellular and antibody mediated rejection in a heart transplant patient with concomitant immense epitope-associated HLA-antibody production: a case report

**DOI:** 10.3389/fimmu.2023.1207373

**Published:** 2023-09-08

**Authors:** Marie Skougaard, Steen Bærentzen, Hans Eiskjær, Pernille Koefoed-Nielsen

**Affiliations:** ^1^ Department of Clinical Immunology, Aarhus University Hospital, Aarhus, Denmark; ^2^ Department of Pathology, Aarhus University Hospital, Aarhus, Denmark; ^3^ Department of Cardiology, Aarhus University Hospital, Aarhus, Denmark

**Keywords:** heart transplantation, mixed rejection, HLA, epitope, antibody, case report

## Abstract

Acute mixed cellular and antibody-mediated rejection (MR) has an estimated prevalence of 7.8%. However, knowledge of MR immune pathogenesis in cardiac graft rejection remains sparse. We report a case of acute MR in a heart transplant patient with a mutation in the MYH7 gene encoding the protein β-myosin heavy chain, resulting in familial hypertrophic cardiomyopathy. The patient presented with substantial eosinophilic infiltration and extensive production of Human Leukocyte Antigen (HLA)-antibodies associated with shared epitopes. Eosinophilic infiltration in the endo- and myocardium was diagnosed in routine post-transplant biopsies stained with hematoxylin-eosin on day 6 after transplantation. On day 27, the patient presented with dyspnea, weight gain, increased pro-brain natriuretic peptide, and was hospitalized due to suspected acute rejection. Endomyocardial biopsies showed eosinophils in endo- and myocardium with additional lymphocytes and hyperplastic endothelium. Immunohistochemistry, including CD31/CD68 double stain confirmed endothelium-associated macrophages in capillaries and severe C4d positivity in the capillaries and endocardial endothelium. Lymphocytes were identified as primarily CD45+/CD3+ T cells with a concomitant few CD45+/CD20+ B cells. HLA-antibody analysis demonstrated a significant increase in 13 HLA-antibodies present in pre-transplant-serum, of which anti-B7 was donor-specific, and 23 strong *de-novo* HLA-class I antibodies of which anti-B62 was donor-specific. 72% of HLA-antibodies, including the two donor-specific antibodies, shared the same HLA antigen epitope; 43P+69A or 163L+167W. This is a case reporting both HLA-antibody and pathohistological data indicating the need for better understanding of interactions between cellular and antibody-mediated immune response mechanisms in graft rejection, and the significance of pre-transplant donor-specific antibodies during immunological pre-transplant risk assessment.

## 1 Introduction

Acute mixed cellular and antibody-mediated rejection (MR) has an estimated prevalence of 7.8% in heart transplant patients (1) in which mild acute cellular rejection (ACR) and antibody-mediated rejection (AMR) often co-exist. However, severe cases are less frequent (2, 3). While ACR is characterized by interstitial lymphocyte T cell infiltration and myocyte injury (4), and AMR by the presence of antibodies against Human Leucocyte Antigens (HLA) (5), the broad definition of acute MR includes cellular infiltrates with concomitant immune pathological traces of AMR in endomyocardial biopsies (2). Nevertheless, the immune pathogenesis of MR in cardiac graft rejection remains poorly understood. Here we present concurrent HLA-antibody and pathohistological data from a case of a heart transplanted patient with acute MR characterized by initial substantial eosinophilic infiltration and immense production of HLA-antibodies associated to two shared HLA epitopes.

## 2 Case description

A Caucasian, 42-year old, female patient diagnosed with MYH7 mutation (6) and familial hypertrophic cardiomyopathy underwent heart transplantation in October 2021. Pre-transplant history included severe left ventricular hypertrophy established on electrocardiogram as a random find in 2005. Thus, indications of cardiac issues during childhood were revealed from patient-derived information. The patient had an implantable cardioverter-defibrillator implanted in 2012 and entered the waiting list for a heart transplant in October 2020 due to decreasing cardiac function, severe symptoms and multiple episodes with malignant ventricular tachycardia.

Routine pre-transplant immunological assessment was performed, including-HLA typing, HLA-antibody identification and immunological risk assessment. Next generation sequencing (NGS, **Omixon Holotype HLA 24/7)** was used for HLA typing on 11 loci. Serum was analyzed for HLA-antibodies using Labscreen Single Antigen ^®^ (One Lambda Inc.) according to the manufacturer’s instructions. Mean fluorescence intensity (MFI) values above 1000 were considered positive. The patient was found to be immunized against HLA-class I. Immunization history prior to transplantation included three children and two blood products received in 2007. The case report was written in accordance with the CARE case report guidelines.

### 2.1 Transplantation

**Figure 1:**
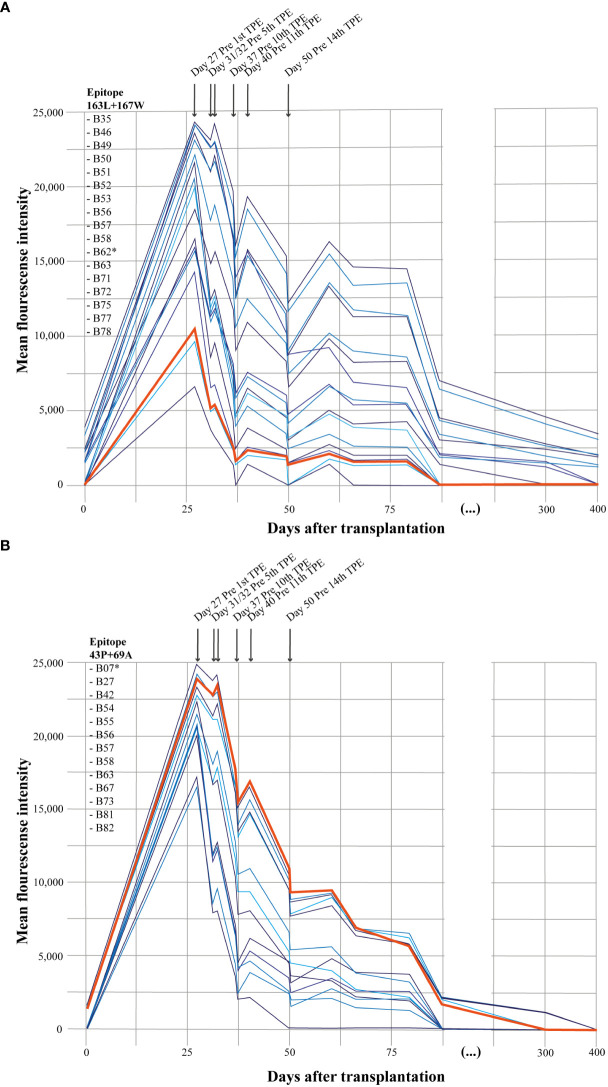
HLA-antibody analysis. Development of HLA-antibodies after heart transplantion grouped by the associated epitope. **(A)** illustrates HLA-antibodies associated with HLA epitope 163L+167W. **(B)** illustrates HLA-antibodies associated with HLA epitope 43P+69A. Donor-specific antibodies; anti-B62 and anti-B7 are marked with a star (*) in the listed HLA-antibodies and the bold orange line in the figure. TPE, terapeutic plasma exchange/plasmapheresis.

Heart transplantation was completed resulting in satisfying post-transplant cardiac function. Immunosuppressive treatment initiated at the time of transplantation included induction therapy with anti-thymocyte globuline (3-day course of 75 mg/day) and methylprednisolone (500 mg before and after extracorporeal circulation, and 120 mg 3 times within the day post-transplant). Maintenance treatment included tacrolimus (2 mg/day), mycophenolate (1000 mg twice daily), and prednisolone (initiating on 25 mg/day). Trough levels of tacrolimus were between 8-14 µg/l for the last 2 weeks before rejection. Patient HLA-type was determined as HLA-A2,24; B60,44; Cw10,5; DR11,13; DQ7,6, while donor HLA-type was determined as HLA-A2; B7,62; Cw1,7; DR4; DQ7,8. The transplantation was performed against a weak donor-specific anti-B7 (**Figure 1**), but a negative flow cytometric T- and B-cell crossmatch.

**Figure 2:**
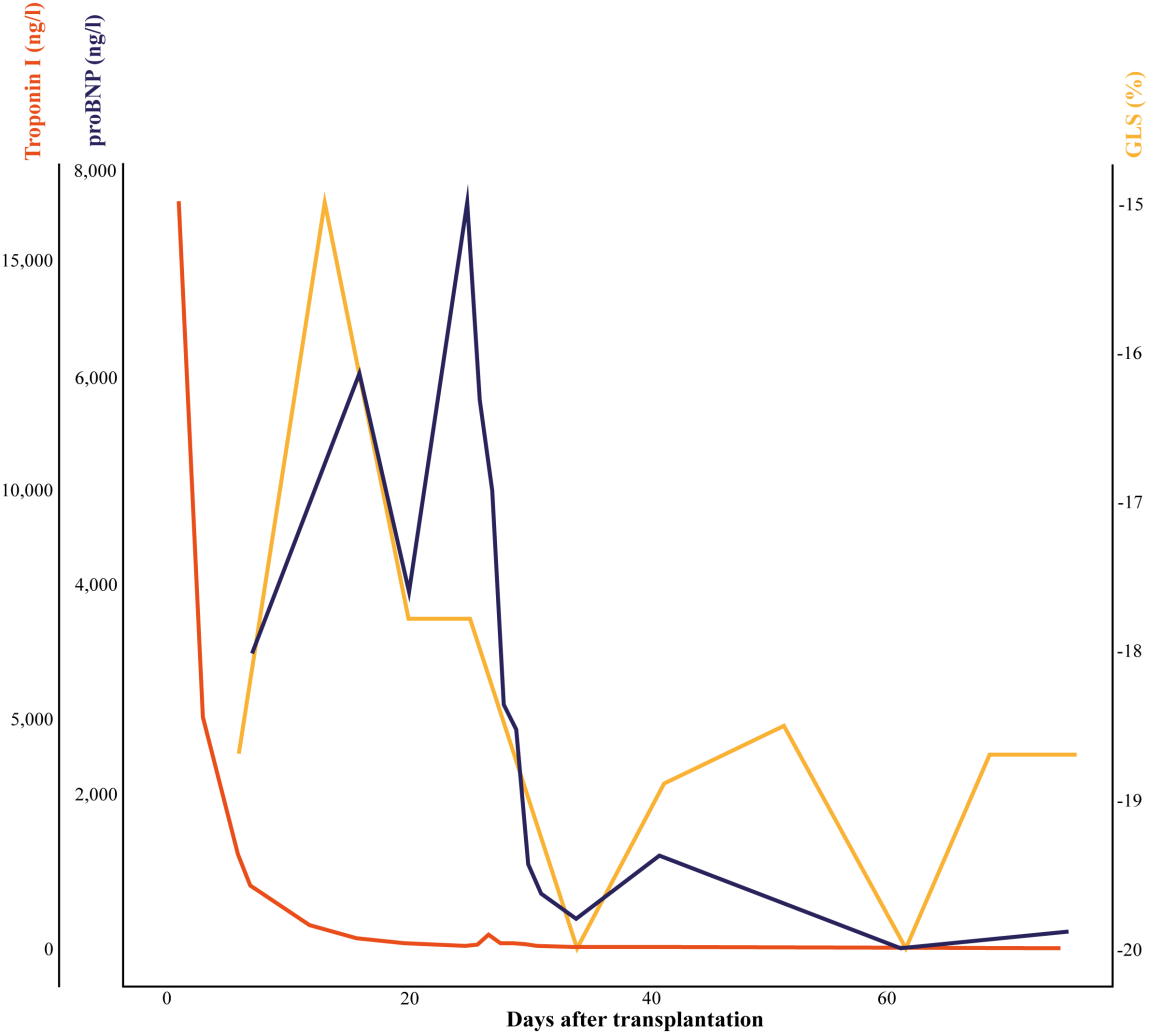
Change in clinical and biochemical measures of cardiac dysfunction. Change in clinical measure, global longitudinal strain, retrieved from transthoracic echocardiogram and biochemical measures, proBNP and troponin I visualizing the development in cardiac dysfunction and myocyte damage from the day of transplantation and to day 75. GLS, global longitudinal strain; proBNP, pro-brain natriuretic peptide.

Post-transplant monitoring included endomyocardial biopsies every week for the first six weeks and planned HLA-antibody screen at week 6 in line with local protocol. Transthoracic echocardiogram was performed at the time of all biopsies retrieving Global Longitudinal Strain (GLS) as a measure of myocardial dysfunction (**Figure 2**). On day 27 after transplantation, the patient presented with dyspnea, weight gain, and increased pro-brain natriuretic peptide (proBNP) of 7,700 ng/l and was hospitalized due to suspected acute rejection. Additional acute endomyocardial biopsy and HLA-antibody identification were conducted. Plasmapheresis was initiated immediately upon hospitalization on day 27 due to the immense increase in HLA-antibodies. The patient received 14 treatments of plasmapheresis completed on day 51 after transplantation. Anti-rejection therapy included Methylprednisolone, Immunoglobulins, and further Rituximab after ended plasmapheresis. Further, basal immunosuppression was intensified. Therapy resulted in normalization of GLS and proBNP (**Figure 2**).

### 2.2 Histology

**Figure 3:**
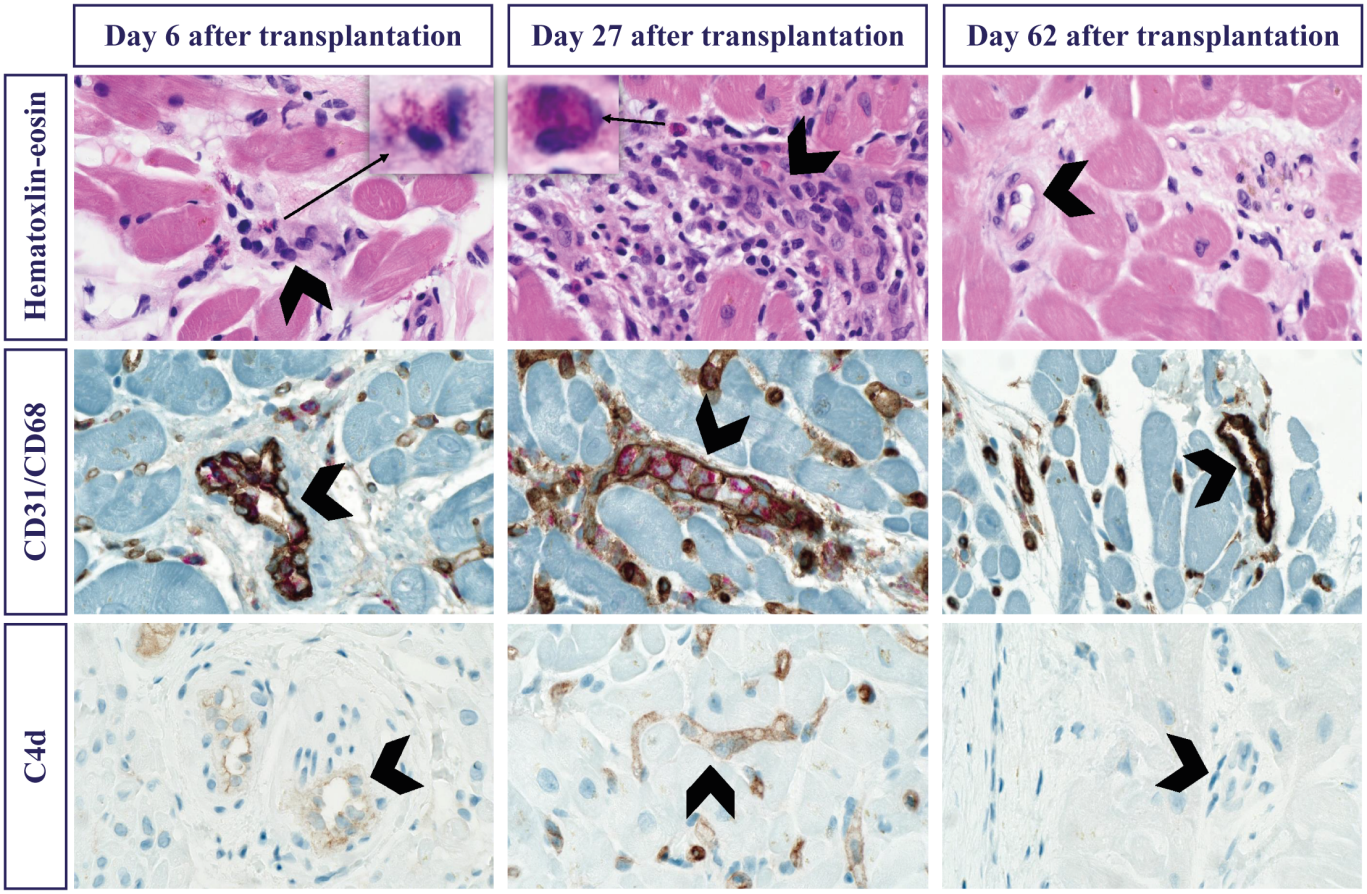
Histological characteristics of endomyocardial biopsies. Biopsies from day 6 (left column), day 27 (center column) and day 62 (right column). Stained with standard Hematoxylin-Eosin (top row), immunohistochemically combined CD31 brown showing endothelial cells and CD68 red showing macrophages (center row) and complement C4D brown (bottom row). Arrows pointing to magnified eosinophilic granulocytes in the interstitium on day 6 and day 27. Arrow heads indicating the most prominent blood vessels with a maximum of macrophages and complement on day 27, having disappeared by day 62.

Standard pathological examination using hematoxylin-eosin stain was performed to evaluate tissue and cellular composition and structure. First biopsy on day 6 after transplantation was graded as 0R with 1 out of 4 fragments showing unspecific endocardial and subendocardial changes in addition to slightly more widespread eosinophilic granulocytes (**Figure 3**). Retrospective immunohistochemistry, including CD31/CD68 and C4d immunostaining, revealed endothelium-associated macrophages in 10% of the capillaries and C4d positivity in 50% of the capillaries and endocardial endothelium, respectively, associated with AMR (5).

At time of hospitalization on day 27, histological traces of graft rejection had worsened, revealing inflammatory areas with substantial infiltration of eosinophils within both endo- and myocardium, concomitant lymphocyte infiltration and hyperplastic endothelium but no signs of myocyte necrosis. Histology revealed no signs of endocardial thrombosis. The CD31/CD68 double stain confirmed interstitial and intraluminal endothelium-associated macrophages in 20% of the capillaries and weak to strong C4d positivity in 100% of the capillaries and endocardial endothelium. Immunohistochemistry defined lymphocytes as primarily CD45+/CD3+ T cells and concomitant few CD45+/CD20+ B cells implying additional ACR-derived mechanisms (**Figure 3**). Histopathological findings were consistent with cellular rejection 1R (2, 4) and humoral rejection pAMR2 (7). Drug hypersensitivity was suspected as an explanation of eosinophilic infiltration. However, the patient was not known to suffer from any allergies nor prior parasite infections and no eosinophils were found in the pathological examination of the explanted heart.

On day 62, after completing 14 plasmapheresis treatments, histopathological examination found no evidence of ongoing rejection. A few lymphocytes were present in the endo- and myocardium in which eosinophils, endothelial hyperplasia and edema were otherwise eradicated. Complement C4d and CD31/CD68 immunostaining were negative (**Figure 3**).

### 2.3 Immunological findings and HLA-antibody status

Peripheral blood leukocytes, including subtypes were within normal range 10 days before transplantation. An initial increase in peripheral leukocytes (16.1x10^9^/L), including monocytes (0.95 x10^9^/L) and neutrophils (14.8 x10^9^/L), together with a decrease in lymphocytes (0.17x10^9^/L) and eosinophils (<0.02 x10^9^/L) were seen in the days after transplantation. The changes were associated to medical induction therapy. At the day of hospitalization peripheral blood eosinophils (0.18 x10^9^/L), lymphocytes (0.68 x10^9^/L) and monocytes (1.02 x10^9^/L) had increased.

HLA-antibody analysis performed on the day of transplantation revealed a weak donor-specific anti-B7 (1,300 MFI) and 12 other HLA class I antibodies. At the day of hospitalization on day 27 after transplantation, pre-existing antibodies had increased significantly and 23 strong *de-novo* HLA-class I antibodies (>6000 MFI) had appeared (**Figure 1**). Among the identified antibodies, two were considered donor-specific antibodies; anti-B7 (identified pre-transplant) and anti-B62 (*de-novo*). Interestingly, 72% of the HLA-antibodies shared the same HLA-epitope; 43P+69A or 163L+167W as donor-specific antibodies anti-B7 and anti-B62 (**Figure 1**), respectively. All HLA-antibodies with MFI>6000 appearing at day 27 were covered by four HLA-epitopes. However, the additional two HLA-epitopes were not associated with known donor-specific antibodies. Intensified anti-rejection therapy, including Rituximab, and plasmapheresis worked to significantly decrease HLA-antibodies (**Figure 1**).

## 3 Discussion

MR has within recent years been assigned a more significant role as it is suggested more frequent than previously considered (8) and associated with a worse prognosis (1, 9). Moreover, MR immune pathogenesis remains poorly understood, and information is sparse on whether cellular and antibody-mediated immune response mechanisms drive graft rejection independently or as co-acting common pathways. Previous observations have implicated eosinophils in allograft rejection (10, 11) which in heart transplants has been associated with more severe rejection (1). This is highly feasible considering the current case report patient and the rejection severity in which eosinophilic infiltration was present in both endo- and myocardium on day 6 after transplantation and most likely before.

Allograft rejection is often associated to the effector function of adaptive immune T- and B-cells. This case report supports the significance of innate cells, including eosinophils and monocytes. Important contribution of both eosinophils and monocytes to cardiac allograft rejection is demonstrated by the combination of increasing number of peripheral blood eosinophils and monocytes, together with histological findings of eosinophils and macrophages, possibly differentiated from emigrated monocytes (12). Standard biochemistry mirrors the relative importance of these cell types with peripheral blood eosinophils being within normal range (0.18 x10^9^/L) and peripheral blood monocytes being just above normal reference range (1.02 x10^9^/L), However, the expansion of peripheral eosinophils and monocytes despite immunosuppressive therapies, associated with increased risk of rejection (13) is considered to reflect the escalating immune activation resulting in recruitment of these innate immune cells to the inflammatory site of the cardiac allograft revealed in the biopsies. Together findings mirror the absolute importance of eosinophils and monocytes in allograft rejection.

The complete elimination of eosinophils (<0.02 x10^9^/L) induced by the large amount of methylprednisolone (14) at day 0 and day 1 after transplantation further imply the possible effect of methylprednisolone during acute allograft rejection eradicating eosinophilic infiltration in the myocardial biopsies. Targeted therapies, including interleukin(IL)-5 inhibitor, have been suggested as treatment of eosinophil associated rejection (15). However, the use of therapies targeting eosinophils in allograft rejection is not established, A reason is the poorly understood immune pathological mechanisms and importance of eosinophils in the development of ACR and AMR, co-occurring as MR.

ACR is somewhat defined as a T cell-mediated process. It has been suggested that the eosinophilic involvement might induce an inflammatory process mediated by T helper cell type 2 (Th2) and Th2-associted cytokines IL-4 and IL-5 (16, 17), possibly occurring in a subgroup of patients suffering from allograft rejection. However, the Th2 polarization has also been suggested to promote amelioration of the inflammatory rejection response (18) which implies the need for further investigation into the importance of eosinophils in allograft rejection and the heterogeneous inflammatory mechanisms, including different immune response pathways leading to allograft rejection. Recipient B cell might be activated by antigen-presenting cells, including both monocytes and T cells, displaying damage-associated molecular patterns (DAMPs) and donor’s foreign antigens (19, 20). Further, Th2-derived cytokines have been implicated in B cell differentiation, activation and establishing an inflammatory milieu influencing the development of antibodies (19).

AMR is characterized by the production of non-HLA and, most often, HLA-antibodies targeting donor HLA-structures produced by B cells (21). The patient presented with HLA-linked AMR with an extensive production of donor-specific and non-donor-specific HLA-antibodies. The increasing pre-existing HLA-antibodies and development of 23 *de-novo* HLA-antibodies were most likely caused by cross-reactivity induced by shared HLA-epitopes (22). HLA-molecules are highly polymorph structures consisting of multiple amino acid epitopes that might be recognized by various specific HLA-antibodies (23). The indication and need for HLA-epitope matching have been discussed previously (22), and evidence presented within the current case report supports the need for additional HLA-epitope matching prior to heart transplantation. Thus, additional research is needed on how to implement HLA-epitope matching during heart transplantation.

Strengths of the case report include that data collected were *real-life* patient data obtained from a heart transplant patient experiencing severe allograft rejection despite standard immunosuppressive treatments. Limitations include limited knowledge and experimental investigation of immune cell functionality, i.e., considering the presence and function of eosinophils, and the functional interactions between innate immune cells and T- and B-cell mediated allograft rejection. Additional experimental studies of cell functionality are needed to improve the understanding of the link innate cells, ACR and AMR, and whether they drive graft rejection independently or as co-acting common pathways leading to MR, which will be our next step.

Based on the successful management and tremendous effect provided by plasmapheresis and medical anti-rejection therapy, i.e., methylprednisolone, Immunoglobulins, and Rituximab, resulting in both significant reduction of HLA-antibodies and elimination of histopathological traces of rejection, it is hypothesized that mechanisms of graft rejection in the current patient is caused by mixed cellular and antibody-mediated rejection. Overall, these observations call for a better understanding of the interplay between cellular and antibody-mediated immune response mechanisms in patients with allograft rejection.

## Data availability statement

The datasets presented in this article are not readily available because of ethical and privacy restrictions. Requests to access the datasets should be directed to the corresponding author/s.

## Ethics statement

According to the Consolidation Act on Research Ethics Review of Health Research Projects, #1338 of 01.09.2020 section 14, only health research studies have to be notified to the Committees. The Central Denmark Region Committees on Health Research Ethics do not consider this study a health research study (section 2). Written informed consent was obtained from the participant/patient(s) for the publication of this case report.

## Author contributions

MS drafted the manuscript. All authors participated in reviewing and editing of the manuscript. SB conducted the pathological microscopic examination. MS was responsible for data analysis and visualization. SB, HE and PK-N provided supervision based on their area of expertise as the responsible pathologist, cardiologist and clinical immunologist, respectively. All authors contributed to the article and approved the submitted version.
